# Use of 9-Valent Human Papillomavirus (HPV) Vaccine: Updated HPV Vaccination Recommendations of the Advisory Committee on Immunization Practices

**Published:** 2015-03-27

**Authors:** Emiko Petrosky, Joseph A. Bocchini, Susan Hariri, Harrell Chesson, C. Robinette Curtis, Mona Saraiya, Elizabeth R. Unger, Lauri E. Markowitz

**Affiliations:** 1 Epidemic Intelligence Service, CDC; 2 National Center for HIV/AIDS, Viral Hepatitis, STD and TB Prevention, CDC; 3 Louisiana State University Health Sciences Center, Shreveport, Louisiana; 4 National Center for Immunization and Respiratory Diseases, CDC; 5 National Center for Chronic Disease Prevention and Health Promotion, CDC; 6 National Center for Emerging and Zoonotic Infectious Diseases, CDC

During its February 2015 meeting, the Advisory Committee on Immunization Practices (ACIP) recommended 9-valent human papillomavirus (HPV) vaccine (9vHPV) (Gardasil 9, Merck and Co., Inc.) as one of three HPV vaccines that can be used for routine vaccination (Table 1). HPV vaccine is recommended for routine vaccination at age 11 or 12 years (1). ACIP also recommends vaccination for females aged 13 through 26 years and males aged 13 through 21 years not vaccinated previously. Vaccination is also recommended through age 26 years for men who have sex with men and for immunocompromised persons (including those with HIV infection) if not vaccinated previously (1). 9vHPV is a noninfectious, virus-like particle (VLP) vaccine. Similar to quadrivalent HPV vaccine (4vHPV), 9vHPV contains HPV 6, 11, 16, and 18 VLPs. In addition, 9vHPV contains HPV 31, 33, 45, 52, and 58 VLPs (2). 9vHPV was approved by the Food and Drug Administration (FDA) on December 10, 2014, for use in females aged 9 through 26 years and males aged 9 through 15 years (3). For these recommendations, ACIP reviewed additional data on 9vHPV in males aged 16 through 26 years (4). 9vHPV and 4vHPV are licensed for use in females and males. Bivalent HPV vaccine (2vHPV), which contains HPV 16, 18 VLPs, is licensed for use in females (1). This report summarizes evidence considered by ACIP in recommending 9vHPV as one of three HPV vaccines that can be used for vaccination and provides recommendations for vaccine use.

*Recommendations for routine use of vaccines in children, adolescents and adults are developed by the Advisory Committee on Immunization Practices (ACIP). ACIP is chartered as a federal advisory committee to provide expert external advice and guidance to the Director of the Centers for Disease Control and Prevention (CDC) on use of vaccines and related agents for the control of vaccine-preventable diseases in the civilian population of the United States. Recommendations for routine use of vaccines in children and adolescents are harmonized to the greatest extent possible with recommendations made by the American Academy of Pediatrics (AAP), the American Academy of Family Physicians (AAFP), and the American College of Obstetricians and Gynecologists (ACOG). Recommendations for routine use of vaccines in adults are harmonized with recommendations of AAFP, ACOG, and the American College of Physicians (ACP). ACIP recommendations approved by the CDC Director become agency guidelines on the date published in the* Morbidity and Mortality Weekly Report *(*MMWR*). Additional information about ACIP is available at http://www.cdc.gov/vaccines/acip/.*

## Methods

From October 2013 to February 2015, the ACIP HPV Vaccine Work Group reviewed clinical trial data assessing the efficacy, immunogenicity, and safety of 9vHPV, modeling data on cost-effectiveness of 9vHPV, and data on burden of type-specific HPV-associated disease in the United States. Summaries of reviewed evidence and Work Group discussions were presented to ACIP before recommendations were proposed. Recommendations were approved by ACIP in February 2015. Evidence supporting 9vHPV use was evaluated using the Grading of Recommendations, Assessment, Development, and Evaluation (GRADE) framework (5) and determined to be type 2 (moderate level of evidence) among females and 3 (low level of evidence) among males; the recommendation was categorized as a Category A recommendation (for all persons in an age- or risk-factor–based group) (6).

## HPV-Associated Disease

HPV is associated with cervical, vulvar, and vaginal cancer in females, penile cancer in males, and anal cancer and oropharyngeal cancer in both females and males (7–10). The burden of HPV infection also includes cervical precancers, including cervical intraepithelial neoplasia grade 2 or 3 and adenocarcinoma in situ (≥CIN2). The majority of all HPV-associated cancers are caused by HPV 16 or 18, types targeted by 2vHPV, 4vHPV and 9vHPV (2,11,12). In the United States, approximately 64% of invasive HPV-associated cancers are attributable to HPV 16 or 18 (65% for females; 63% for males; approximately 21,300 cases annually) and 10% are attributable to the five additional types in 9vHPV: HPV 31, 33, 45, 52, and 58 (14% for females; 4% for males; approximately 3,400 cases annually) (1,12,13). HPV 16 or 18 account for 66% and the five additional types for about 15% of cervical cancers (12). Approximately 50% of ≥CIN2 are caused by HPV 16 or 18 and 25% by HPV 31, 33, 45, 52, or 58 (14). HPV 6 or 11 cause 90% of anogenital warts (condylomata) and most cases of recurrent respiratory papillomatosis (15).

## 9vHPV Efficacy, Immunogenicity, and Safety

In a phase III efficacy trial comparing 9vHPV with 4vHPV among approximately 14,000 females aged 16 through 26 years, 9vHPV efficacy for prevention of ≥CIN2, vulvar intraepithelial neoplasia grade 2 or 3, and vaginal intraepithelial neoplasia grade 2 or 3 caused by HPV 31, 33, 45, 52, or 58 was 96.7% in the per protocol population* (Table 2) (2,16). Efficacy for prevention of ≥CIN2 caused by HPV 31, 33, 45, 52, or 58 was 96.3% and for 6-month persistent infection was 96.0% (16). Few cases were caused by HPV 6, 11, 16, or 18 in either vaccine group. Noninferior immunogenicity of 9vHPV compared with 4vHPV was used to infer efficacy for HPV 6, 11, 16, and 18. Geometric mean antibody titers (GMTs) 1 month after the third dose were noninferior for HPV 6, 11, 16, and 18; in the 9vHPV group, >99% seroconverted to all nine HPV vaccine types (Table 3).

Two immunobridging trials were conducted. One compared 9vHPV in approximately 2,400 females and males aged 9 through 15 years with approximately 400 females aged 16 through 26 years. Over 99% seroconverted to all nine HPV vaccine types; GMTs were significantly higher in adolescents aged 9 through 15 years compared with females aged 16 through 26 years. In a comparison of 4vHPV with 9vHPV in approximately 600 adolescent females aged 9 through 15 years, 100% seroconverted to HPV 6, 11, 16, and 18 in both groups, and GMTs were noninferior in the 9vHPV group compared with the 4vHPV group.

Immunogenicity in males aged 16 through 26 years was compared with females of the same age group in a separate study. In both females and males, >99% seroconverted to all nine HPV vaccine types, and GMTs in males were noninferior to those in females (4).

The immunogenicity of concomitant and nonconcomitant administration of 9vHPV with quadrivalent meningococcal conjugate vaccine (Menactra, MenACWY-D) and tetanus, diphtheria, acellular pertussis vaccine (Adacel, Tdap) was evaluated. The GMTs were noninferior for all nine HPV vaccine types in the co-administered group (all p<0.001). For Menactra, the noninferiority criterion was met for all four serogroups, and for Adacel, for diphtheria, tetanus, and all four pertussis antigens.

Safety has been evaluated in approximately 15,000 subjects in the 9vHPV clinical development program; approximately 13,000 subjects in six studies were included in the initial application submitted to FDA (2). The vaccine was well-tolerated, and most adverse events were injection site-related pain, swelling, and erythema that were mild to moderate in intensity. The safety profiles were similar in 4vHPV and 9vHPV vaccinees. Among females aged 9 through 26 years, 9vHPV recipients had more injection-site adverse events, including swelling (40.3% in the 9vHPV group compared with 29.1% in the 4vHPV group) and erythema (34.0% in the 9vHPV group compared with 25.8% in the 4vHPV group). Males had fewer injection site adverse events. In males aged 9 through 15 years, injection site swelling and erythema in 9vHPV recipients occurred in 26.9% and 24.9%, respectively. Rates of injection-site swelling and erythema both increased following each successive dose of 9vHPV.

## Health Impact and Cost Effectiveness

Introduction of 9vHPV in both males and females was cost-saving when compared with 4vHPV for both sexes in a cost-effectiveness model that assumed 9vHPV cost $13 more per dose than 4vHPV. Cost-effectiveness ratios for 9vHPV remained favorable compared with 4vHPV (9vHPV was cost-saving in most scenarios, and the cost per quality-adjusted life year gained did not exceed $25,000 in any scenario) when varying assumptions about HPV natural history, cervical cancer screening, vaccine coverage, vaccine duration of protection, and health care costs, but were sensitive to 9vHPV cost assumptions (17). Because the additional five types in 9vHPV account for a higher proportion of HPV-associated cancers in females compared with males and cause cervical precancers, the additional protection from 9vHPV will mostly benefit females.

## Recommendations for Use of HPV Vaccines

ACIP recommends that routine HPV vaccination be initiated at age 11 or 12 years. The vaccination series can be started beginning at age 9 years. Vaccination is also recommended for females aged 13 through 26 years and for males aged 13 through 21 years who have not been vaccinated previously or who have not completed the 3-dose series (1). Males aged 22 through 26 years may be vaccinated.† Vaccination of females is recommended with 2vHPV, 4vHPV (as long as this formulation is available), or 9vHPV. Vaccination of males is recommended with 4vHPV (as long as this formulation is available) or 9vHPV.

2vHPV, 4vHPV, and 9vHPV all protect against HPV 16 and 18, types that cause about 66% of cervical cancers and the majority of other HPV-attributable cancers in the United States (1,12). 9vHPV targets five additional cancer causing types, which account for about 15% of cervical cancers (12). 4vHPV and 9vHPV also protect against HPV 6 and 11, types that cause anogenital warts.

**What is currently recommended?**

The Advisory Committee on Immunization Practices (ACIP) recommends routine HPV vaccination at age 11 or 12 years. The vaccination series can be started beginning at age 9 years. Vaccination is also recommended for females aged 13 through 26 years and for males aged 13 through 21 years who have not been vaccinated previously or who have not completed the 3-dose series. Males aged 22 through 26 years may be vaccinated. ACIP recommends vaccination of men who have sex with men and immunocompromised persons through age 26 years if not vaccinated previously.

**Why are the recommendations being updated now?**

9-valent HPV vaccine (9vHPV) was approved by the Food and Drug Administration on December 10, 2014. This vaccine targets HPV types 6, 11, 16, and 18, the types targeted by the quadrivalent HPV vaccine (4vHPV), as well as five additional types, HPV types 31, 33, 45, 52, and 58. ACIP reviewed results of a randomized trial among approximately 14,000 females aged 16 through 26 years that showed noninferior immunogenicity for the types shared by 4vHPV and 9vHPV and high efficacy for the five additional types. Other trials in the 9vHPV clinical development program included studies that compared antibody responses across age groups and females and males and concomitant vaccination studies. The evidence supporting 9vHPV vaccination was evaluated using the Grading of Recommendations, Assessment, Development, and Evaluation (GRADE) framework and determined to be type 2 (moderate level of evidence) among females and 3 (low level of evidence) among males; the recommendation was designated as a Category A recommendation (recommendation for all persons in an age- or risk-factor–based group).

**What are the new recommendations?**

9vHPV, 4vHPV or 2vHPV can be used for routine vaccination of females aged 11 or 12 years and females through age 26 years who have not been vaccinated previously or who have not completed the 3-dose series. 9vHPV or 4vHPV can be used for routine vaccination of males aged 11 or 12 years and males through age 21 years who have not been vaccinated previously or who have not completed the 3-dose series. ACIP recommends either 9vHPV or 4vHPV vaccination for men who have sex with men and immunocompromised persons (including those with HIV infection) through age 26 years if not vaccinated previously.

### Administration

2vHPV, 4vHPV, and 9vHPV are each administered in a 3-dose schedule. The second dose is administered at least 1 to 2 months after the first dose, and the third dose at least 6 months after the first dose§ (1). If the vaccine schedule is interrupted, the vaccination series does not need to be restarted.

If vaccination providers do not know or do not have available the HPV vaccine product previously administered, or are in settings transitioning to 9vHPV, any available HPV vaccine product may be used to continue or complete the series for females for protection against HPV 16 and 18; 9vHPV or 4vHPV may be used to continue or complete the series for males. There are no data on efficacy of fewer than 3 doses of 9vHPV.

### Special Populations

HPV vaccination is recommended through age 26 years for men who have sex with men and for immunocompromised persons (including those with HIV infection) who have not been vaccinated previously or have not completed the 3-dose series.

### Precautions and Contraindications

HPV vaccines are contraindicated for persons with a history of immediate hypersensitivity to any vaccine component. 4vHPV and 9vHPV are contraindicated for persons with a history of immediate hypersensitivity to yeast. 2vHPV should not be used in persons with anaphylactic latex allergy.

HPV vaccines are not recommended for use in pregnant women (1). If a woman is found to be pregnant after initiating the vaccination series, the remainder of the 3-dose series should be delayed until completion of pregnancy. Pregnancy testing is not needed before vaccination. If a vaccine dose has been administered during pregnancy, no intervention is needed. A new pregnancy registry has been established for 9vHPV (2). Pregnancy registries for 4vHPV and 2vHPV have been closed with concurrence from FDA (1,18). Exposure during pregnancy can be reported to the respective manufacturer.¶ Patients and health care providers can report an exposure to HPV vaccine during pregnancy to the Vaccine Adverse Event Reporting System (VAERS).

Adverse events occurring after administration of any vaccine should be reported to VAERS. Additional information about VAERS is available by telephone (1–800–822–7967) or online at http://vaers.hhs.gov.

### Cervical Cancer Screening

Cervical cancer screening is recommended beginning at age 21 years and continuing through age 65 years for both vaccinated and unvaccinated women (19,20). Recommendations will continue to be evaluated as further postlicensure monitoring data become available.

## Future Policy Issues

A clinical trial is ongoing to assess alternative dosing schedules of 9vHPV. ACIP will formally review the results as data become available. HPV vaccination should not be delayed pending availability of 9vHPV or of future clinical trial data.

## Figures and Tables

**TABLE 1:** Characteristics of the three human papillomavirus (HPV) vaccines licensed for use in the United States

Characteristic	Bivalent (2vHPV)*	Quadrivalent (4vHPV)†	9-valent (9vHPV)§
Brand name	Cervarix	Gardasil	Gardasil 9
VLPs	16, 18	6, 11, 16, 18	6, 11, 16, 18, 31, 33, 45, 52, 58
Manufacturer	GlaxoSmithKline	Merck and Co., Inc.	Merck and Co., Inc.
Manufacturing	*Trichoplusia ni* insect cell line infected with L1 encoding recombinant baculovirus	*Saccharomyces cerevisiae* (Baker’s yeast), expressing L1	*Saccharomyces cerevisiae* (Baker’s yeast), expressing L1
Adjuvant	500 *μ*g aluminum hydroxide, 50 *μ*g 3-O-desacyl-4′ monophosphoryl lipid A	225 *μ*g amorphous aluminum hydroxyphosphate sulfate	500 *μ*g amorphous aluminum hydroxyphosphate sulfate
Volume per dose	0.5 ml	0.5 ml	0.5 ml
Administration	Intramuscular	Intramuscular	Intramuscular

**Abbreviation:** L1 = the HPV major capsid protein; VLPs = virus-like particles.

* Only licensed for use in females in the United States. Package insert available at http://www.fda.gov/downloads/BiologicsBloodVaccines/Vaccines/ApprovedProducts/UCM186981.pdf.

† Package insert available at http://www.fda.gov/downloads/BiologicsBloodVaccines/Vaccines/ApprovedProducts/UCM111263.pdf.

§ Package insert available at http://www.fda.gov/downloads/BiologicsBloodVaccines/Vaccines/ApprovedProducts/UCM426457.pdf.

**TABLE 2:** Results of a Phase III efficacy trial comparing 9-valent human papillomavirus (HPV) vaccine (9vHPV) with quadrivalent HPV vaccine (4vHPV), per protocol population* in females aged 16 through 26 years†

		9vHPV		4vHPV		Vaccine efficacy	
				
Endpoint-related types	Endpoint	No. participants	Cases	No. participants	Cases	%	(95% CI)
HPV 31, 33, 45, 52, 58	≥CIN2, VIN2/3, VaIN2/3	6,016	1	6,017	30	96.7	(80.9–99.8)
	≥CIN2	5,948	1	5,943	27	96.3	(79.5–99.8)
	6-month persistent infection	5,939	35	5,953	810	96.0	(94.4–97.2)
HPV 6, 11, 16, 18	≥CIN2^§^	5,823	1	5,832	1	—	—
	Anogenital warts	5,876	5	5,893	1	—	—

**Abbreviations:** CI = confidence interval; ≥CIN2 = cervical intraepithelial neoplasia grade 2 or 3 or adenocarcinoma in situ; VaIN2/3 = vaginal intraepithelial neoplasia grade 2 or 3; VIN2/3 = vulvar intraepithelial neoplasia grade 2 or 3.

**Sources:** Package insert available at http://www.fda.gov/downloads/BiologicsBloodVaccines/Vaccines/ApprovedProducts/UCM426457.pdf. Joura EA, Giuliano AR, Iversen OE, et al. A 9-valent HPV vaccine against infection and intraepithelial neoplasia in women. N Engl J Med 2015;372:711–23.

* Females who received all 3 vaccinations within 1 year of enrollment, did not have major deviations from the study protocol, were naïve (polymerase chain reaction [PCR] negative and seronegative) to the relevant HPV type(s) before dose 1, and who remained PCR negative to the relevant HPV type(s) through 1 month after dose 3 (month 7).

† Participants were enrolled from sites in 18 countries; median duration of follow-up was 40 months.

**TABLE 3:** Human papillomavirus (HPV) 6, 11, 16, and 18 seroconversion and geometric mean titers (GMTs*) after 3 doses of 9-valent HPV vaccine (9vHPV) compared with quadrivalent HPV vaccine (4vHPV), per protocol population† in females aged 16 through 26 years§

	9vHPV			4vHPV		
		
Assay (cLIA)	No. participants	Seropositivity (%)	GMT (mMU/mL)	No. participants	Seropositivity (%)	GMT (mMU/mL)
Anti-HPV 6	3,993	(99.8)	893	3,975	(99.8)	875
Anti-HPV 11	3,995	(100)	666	3,982	(99.9)	830
Anti-HPV 16	4,032	(100)	3,131	4,062	(100)	3,157
Anti-HPV 18	4,539	(99.8)	805	4,541	(99.7)	679

**Abbreviations:** cLIA = competitive Luminex immunoassay; mMU = milli-Merck units.

**Source:** Joura EA, Giuliano AR, Iversen OE, et al. A 9-valent HPV vaccine against infection and intraepithelial neoplasia in women, and supplementary appendix. N Engl J Med 2015;372:711–23.

* The noninferiority criterion for GMTs was met for all four HPV types (p<0.001).

† Females who received all 3 vaccinations within 1 year of enrollment, did not have major deviations from the study protocol, were naïve (polymerase chain reaction [PCR] negative and seronegative) to the relevant HPV type(s) before dose 1, and who remained PCR–negative to the relevant HPV type(s) through 1 month after dose 3 (month 7).

§ Participants were enrolled from sites in 18 countries; median duration of follow-up was 40 months.

## Footnotes

* Females who received all 3 vaccinations within 1 year of enrollment, did not have major deviations from the study protocol, were naïve (polymerase chain reaction [PCR] negative and seronegative) to the relevant HPV type(s) before dose 1, and who remained PCR negative to the relevant HPV type(s) through 1 month after dose 3 (month 7).

† Vaccination is also recommended through age 26 years for men who have sex with men and for immunocompromised persons (including those with HIV infection) if not vaccinated previously.

§ Minimum intervals are 1 month between the first and second dose, 3 months between the second and third dose, and 6 months between the first and third dose.

¶ 9vHPV exposure during pregnancy should be reported to the Merck Pregnancy Registry at telephone 1-800-986-8999; 4vHPV exposure during pregnancy can be reported to Merck at telephone 1-877-888-4231. 2vHPV exposure during pregnancy can be reported to GlaxoSmithKline at telephone 1-888-825-5249.
